# Individual and combined effects of acute delta-9-tetrahydrocannabinol and cannabidiol on psychotomimetic symptoms and memory function

**DOI:** 10.1038/s41398-018-0191-x

**Published:** 2018-09-05

**Authors:** Celia J. A. Morgan, Tom P. Freeman, Chandni Hindocha, Grainne Schafer, Chelsea Gardner, H. Valerie Curran

**Affiliations:** 1 Psychopharmacology and Addiction Research Centre, University of Exeter, Exeter, UK; 2 Clinical Psychopharmacology Unit, University College London, London, UK

## Abstract

The main active ingredient in cannabis, delta-9-tetrahydrocannabinol (THC), can acutely induce psychotic symptoms and impair episodic and working memory. Another major constituent, cannabidiol (CBD), may attenuate these effects. This study aimed to determine the effects of THC and CBD, both alone and in combination on psychotic symptoms and memory function. A randomised, double-blind crossover design compared the effects of (i) placebo, (ii) THC 8 mg, (iii) CBD 16 mg and (iv) THC 8 mg + CBD 16 mg administered by inhalation through a vaporiser. Using an experimental medicine approach to predict treatment sensitivity, we selected 48 cannabis users from the community on the basis of (1) schizotypal personality questionnaire scores (low, high) and (2) frequency of cannabis use (light, heavy). The Brief Psychiatric Rating Scale (BPRS), Psychotomimetic States Inventory (PSI), immediate and delayed prose recall (episodic memory), 1- and 2-back (working memory) were assessed on each day. Results indicated that THC increased overall scores on the PSI, negative symptoms on BPRS, and robustly impaired episodic and working memory. Co-administration of CBD did not attenuate these effects. CBD alone reduced PSI scores in light users only. At a ratio of 2:1, CBD does not attenuate the acute psychotic and memory impairing effects of vaporised THC. Frequent cannabis users may show a blunted anti- psychotic response to CBD, which is of concern due to the high rates of cannabis use disorders in patients with schizophrenia.

## Introduction

Cannabis (marijuana) is used by over 180 million people worldwide^1^. Possible consequences of use include dependency, cognitive impairment and increased risk of psychotic illness^2^. However, most people who try cannabis do not experience prolonged adverse effects. Several factors predict vulnerability, including the rs2494732 locus of the AKT1 genotype^3–5^, adolescent exposure^6,7^, frequency of use^8–10^, schizotypy or schizophrenia^11–15^ and the type of cannabis used^2,16^. Although it is typically classified as a single drug, the cannabis plant can contains over 100 unique ‘cannabinoids’, with diverse and sometimes opposing pharmacological actions^17^.

Cannabis containing high levels of delta-9-tetrahydrocannabinol (THC) and little if any cannabidiol (CBD) is becoming increasingly prevalent^18,19^ and is linked to greater cannabis dependency, memory impairment and paranoia^20^ and increased risk of psychotic illness^21^.

Delta-9-THC produces the effects that users seek from cannabis, including ‘stoned’, ‘like drug effect’ and ‘want more drug’^22,23^. THC elicits robust, dose-dependent impairments in immediate and delayed verbal memory^2,24^ and transient positive and negative symptoms reminiscent of schizophrenia^25,26^.

CBD is non-intoxicating and does not influence ratings of ‘stoned’ following the administration of THC or cannabis^2,16^. However, CBD can produce opposite effects to THC across a range tasks and functional neuroimaging assessments^26–28^. In terms of behavioural effects, CBD given alone was found to improve memory consolidation^29^ and in combination with THC is associated with higher recognition memory scores in chronic cannabis users^30^. CBD also appeared to block the impairing effects of THC on verbal recall in a naturalistic study^31^, which was replicated in a laboratory study of oral CBD and intravenous THC^32^.

In terms of psychosis, CBD displayed equivalent efficacy to a standard antipsychotic drug for the treatment of positive and negative symptoms^33^. A preliminary study with 6 volunteers found that oral CBD pre-treatment reduced acute psychotic symptoms following intravenous THC^26^. In a subsequent study of 48 volunteers, CBD reduced the incidence of clinically significant positive psychotic symptoms (but not their overall severity) following intravenous THC^32^. However, a naturalistic study did not find evidence for protective effects of CBD on THC-induced psychotic-like symptoms^31^.

Chronic exposure to CBD has also been linked to fewer psychotic-like symptoms in those who have been exposed to THC^34^, a finding that was replicated in light but not heavy cannabis users^30^.

Taken together, the available data provides some evidence that CBD protects against the harmful effects of THC on memory and psychotic-like symptoms. However, only one study has examined the interactive effects of inhaled THC and CBD^35,36^ no study to our knowledge has examined the interactive effects of THC and CBD on these variables in an experimental design with an inhaled route of administration^37^, which better reflects how cannabis is typically administered than oral or intravenous routes. Furthermore, preliminary data suggest that frequency of cannabis use and schizotypy may predict how an individual responds to THC and and/or CBD. However, we are unaware of any experimental studies comparing the effects of different cannabinoid combinations (e.g. THC, THC + CBD, CBD) among volunteers selected for their vulnerability or resilience.

Here, we adopted an experimental medicine approach in order to assess the effects of cannabinoids on psychotic-like symptoms and memory function. A randomised, double- blind, crossover design was used to mimic the effects of cannabis with varying cannabinoid concentrations, as well as CBD alone. Across four sessions, each volunteer received THC (8 mg), THC (8 mg) + CBD (16 mg), CBD (16 mg) and placebo (ethanol vehicle). We predicted, firstly, that memory impairment and psychotic-like symptoms would occur following THC, secondly, CBD would offset these effects when co- administered with THC, and thirdly, CBD alone would have pro-cognitive and anti- psychotic effects. In order to extend previous work showing that schizotypy/psychosis and cannabis use frequency are possible vulnerability/resilience factors cannabis users were selected from a large-scale study on the basis of their cannabis use and schizotypal personality scores^30^. We predicted that infrequent users^8–10,30^ and people with high psychosis proneness^11,12,14,15^ would show heightened susceptibility to THC, CBD, and their interactive effects.

## Methods

### Participants

Participants were recruited on the basis of having previously volunteered in a large scale study of over 400 cannabis users^30^. Those scoring in the top and bottom quartiles of (1) Schizotypal Personality Questionnaire score (low, high) were invited to take part, from this group we set out to recruit 24 light (1–24 days per month) and 24 heavy (25 + days per month) cannabis users. Additional data from this study on facial affect recognition and effects on a visual analogue scale have been reported elsewhere^38^.

Subjects were matched for age and estimated premorbid verbal intelligence (as measured by the Spot the Word task^39^) across heavy and light users. Inclusion criteria were: (i) self-reported abstinence from cannabis, other drugs and alcohol use for 24 h prior to each test day; (ii) fluent in English, (iii) normal or corrected to normal vision.

Exclusion criteria were: current self-reported (i) respiratory health problems or physical health problems, (ii) pregnancy or the risk of being pregnant, (iii) clinically diagnosed learning impairments, (iv) clinically diagnosed schizophrenia/psychosis or substance abuse problems and (v) no illicit drug use other than cannabis more than once a week.

### Design

A four session, randomised, double-blind crossover design was used to compare the acute effects of THC (8 mg), CBD (16 mg) and their combination (8 mg THC + 16 mg CBD) with placebo (ethanol vehicle). Both cannabinoids were formulated in alcohol solution and were purchased from STI Pharmaceuticals (Brentwood, Essex, UK). A total of 48 volunteers completed the study, comprised equally from the following groups: low schizotypy, light cannabis users (LS-L); low schizotypy, heavy users (LS-H); high schizotypy, light users (HS-L); high schizotypy, heavy users (HS-H). *N* = 12 per experimental group was chosen to detect THC-induced (compared to placebo) impairment in memory at a power of 0.83^22^. Treatment order across the 4 sessions was determined by a balanced Latin square.

#### Procedure

Experimental sessions occurred on four occasions each separated by a one-week wash-out to minimise carry-over effects ( > 3 times elimination half-life of THC^25^). We used urine and saliva screens to verify drug use. Participants completed baseline assessments before, and then commencing 10 min after drug administration. The full test battery took approximately 1.5 h on each test day. Participants were reimbursed £120 for their time on the last testing day and debriefed fully. All participants provided written, informed consent on each occasion and ethical approval was given by the UCL Research Ethics Committee.

### Drug administration

Cannabinoids and placebo (ethanol vehicle) were administered using a Volcano Medic Vaporisor (Storz & Bickel, Tuttlingen, Germany). 8 mg THC dissolved in ethanol and 16 mg of CBD dissolved in ethanol^38^ were administered on a 10-s inhalation cycle wherein participants was instructed to first fully exhale, next fully inhale from the balloon, hold their breath for 10 s and then fully exhale; this was repeated until the balloon was empty^40^. This inhaled dose of THC has been found to produce effects on human brain and behaviour, including psychotic-like symptoms and memory impairment^7,40,41^. The 2:1 ratio of CBD:THC reflects the upper limit (mean + 3 SD) found in high CBD/low THC cannabis preparations^42^. Participants were given a test balloon to familiarise themselves with the procedure before any drug administration occurred. The balloon was filled, covered with an opaque bag, and administered by an independent researcher so that the experimenter collecting behavioural data and participant was blind to drug condition.

### Assessments

Before drug administration participants completed the Beck Depression Inventory^43^, Spielberger Trait Anxiety Inventory^44^, Schizotypal Proneness Questionnaire^45^ and Spot the Word Test^39^. After drug administration, the 48-item Psychotomimetic States Inventory^46^ was used to assess acute schizotypal symptoms. It has subscales of perceptual distortion, cognitive disorganisation, anhedonia, mania, paranoia and delusionary thinking. Each item is rated from 0 (not at all) to 3 (strongly) on statements describing current experiences. Current psychiatric symptoms were assessed with the experimenter-rated Brief Psychiatric Rating Scale^47^ rated from 0 (not present) to 7 (extremely severe) with subscales of positive symptoms and negative symptoms. Participants also completed cognitive measures post-drug administration.

### Cognitive measures

#### Prose recall

Verbal memory was assessed using immediate and delayed prose recall^48^. Participants were required to recall a short passage of prose (30 s news bulletin) immediately and after a 20 min delay filled with other assessments. 4 versions of the prose recall were administered in a counterbalanced order.

#### N-back

This task taps spatial working memory with an increasing load. It has previously shown sensitivity to acute^49^ and chronic^50^ drug effects. The participant was presented with a symbol (smiley face) in one of six spatial locations. A fixation cross remained in the centre of the screen throughout the task. When the next face appeared, they were required to indicate whether it was in the same location as the previous face in the 1- back version of the task, or the same location as the face two positions before (2-back). Each block consisted of 25 “match” and 25 “no-match” trials in random order, i.e. 50 trials in total, preceded by ten practice trials. All symbols were presented 5 cm from the fixation cross. Each symbol was presented for 300 ms with an inter-stimulus interval (ISI) of 450 msec. Versions were randomised across testing days.

#### Fluency (57)

To assess phonological and sematic fluency respectively, participants were asked to generate as many words as possible in 60 s starting with a pre-determined letter, or exemplars related to a pre-determined category. On each testing day participants generated exemplars of one letter and one category.

#### Reitan’s trailmaking test (TMT: 59)

Processing speed was measured using the TMT (Form A and B). Form A requires participants to connect 25 numbers in an ascending numerical sequence. Form B requires participants to connect 13 numbers (1–13) and 12 letters (A-L) in an ascending number-letter sequence. The dependent variable is time to complete the task, and then the time to complete the B form subtracting the basic psychomotor speed (B-A).

### Statistical analysis

Data were analysed using IBM SPSS version 20.

Demographics and scores on questionnaires were analysed using repeated measures ANOVAs with two between-subjects variables (frequency of use, schizotypy).

Assumptions of parametric tests were examined and data were transformed where they were not normally distributed, however in practice this did not alter the outcome so the of the analyses of the untransformed data are reported. Drug was entered as a within subjects factor, and was coded as a simple contrast (Placebo versus THC, Placebo versus THC + CBD, Placebo versus CBD). Additional within subjects factors were added where appropriate (Subscale for the Psychotomimetic States Inventory and Brief Psychiatric Rating Scale, Delay for prose recall, Load for N-back). Interactions with Drug were explored using simple contrasts. Interactions between other factors in repeated measures ANOVA models were analysed using pairwise comparisons with a local Bonferroni correction. Pearson correlational analyses were performed to explore the impact of frequency of cannabis use on any drug effects, and were also Bonferroni-corrected. All statistical tests were two-tailed and *p* values are displayed uncorrected in the text.

## Results

### Group characteristics

As shown in Table 1, groups did not differ in age (*F*_(3,44)_ = 2.540, *p* = 0.069), gender (*X*^2^_(3)_ = 4.437, *p* = 0.218), years of education (*F*_(3,44)_ = 1.575, *p* = 0.209), scores on the spot the word task (*F*_(3,44)_ = 0.802, *p* = 0.499), last use of cannabis (*F*_(3,44)_ = 1.223, *p* = 0.313), or number of years cannabis had been used (*F*_(3,44)_ = 0.666, *p* = 0.578). BDI scores were missing for two participants (heavy user, low schizotypy group) and were replaced with the group mean. There was a main effect of schizotypy on scores on the SPQ (*F*_(1,44)_ = 12.473, *p* = 0.001), BDI (*F*_(1,44)_ = 14.989, *p* < 0.001) and STAI (*F*_(1,44)_ = 9.054, *p* = 0.004) where the high schizotypy group had higher scores than the low schizotypy group for each measure. Light and heavy users of cannabis differed on the time to smoke a standard quantity of cannabis sold in the UK (3.5 g; 1/8 oz) (*F*_(1,44)_ = 8.539, *p* = 0.005) and on the number of days per month they used cannabis (*F*_(1,44)_ = 32.295, *p* < 0.001) where heavy users smoked 3.5 g in fewer days, and used cannabis on more days per month than light users. There were no differences in the number of people who had used tobacco (*X*^2^_(3)_ = 4.457, *p* = 0.208), days since last use of tobacco (*F*_(3,33)_ = 0.592, *p* = 0.625), years of tobacco use (*F*_(3,31)_ = 0.352, *p* = 0.788), or days per month of tobacco use (*F*_(3,31)_ = 0.688, *p* = 0.566). For alcohol, no differences were found for years used (*F*_(3,44)_ = 0.207, *p* = 0.891) or days per month of use (*F*_(3,44)_ = 0.693, *p* = 0.561).

**Table 1:** Means (SD) for demographic, mental health, and drug use variables for light and heavy cannabis users

	Light		Heavy	
	Low schizotypy	High schizotypy	Low schizotypy	High schizotypy
Age	21.00 (2.13)	22.90 (2.02)	21.42 (1.62)	21.5 (1.38)
Gender ratio (m:f)	9:3	7:5	11:1	7:5
Education (years)	15.75 (1.22)	15.79 (1.30)	15.04 (1.77)	14.5 (2.31)
BDI-11	3.25 (3.91)	7.67 (7.10)	3.00 (1.70)	15.75 (12.96)
SPQ	9.25 (12.66)	22.83 (11.84)	10.58 (7.07)	22.8 (17.07)
STAI	35.67 (10.29)	41.67 (8.19)	33.00 (6.63)	42.58 (10.25)
Spot the word task	51.17 (5.13)	49.75 (4.37)	51.42 (4.89)	48.75 (4.94)
Cannabis (*N*)	12	12	12	12
Cannabis used (years)	5.88 (3.48)	6.91 (3.00)	5.92 (2.15)	5.33 (2.39)
Cannabis use (days/month)	11.92 (6.84)	11.71 (10.24)	24.38 (9.06)	26.00 (5.64)
Days since last use	2.50 (1.38)	13.83 (33.64)	4.66 (8.15)	1.92 (0.79)
Time to smoke 3.5 g (days)	11.50 (15.83)	20.54 (16.13)	7.52 (8.84)	3.92 (2.75)
Alcohol (*N*)	12	12	12	12
Alcohol used (years)	6.04 (2.18)	6.71 (2.66)	6.5 (2.19)	5.25 (7.85)
Alcohol (days/month)	11.54 (5.66)	8.04 (4.87)	10.00 (7.67)	11.12 (7.43)
Tobacco (*N*)	6	9	10	9
Tobacco used (years)	4.57 (1.90)	5.22 (2.54)	5.5 (2.37)	5.83 (3.02)
Tobacco (days/month)	20.00 (11.40)	22.45 (12.16)	23.8 (10.89)	27.56 (7.33)
Tobacco cigarettes/day	6.66 (3.77)	6.39 (3.12)	8.55 (5.31)	9.22 (4.47)

### Psychotomimetic states inventory (PSI)

There was a main effect of Drug (*F*_(2,105)_ = 5.550, *p* = 0.003, *ŋ*_*p*_^*2*^ = 0.112), driven by increased scores relative to placebo for THC (*p* = 0.014) and THC + CBD (*p* = 0.022) but no change following CBD (*p* = 0.544).

**Fig. 1:**
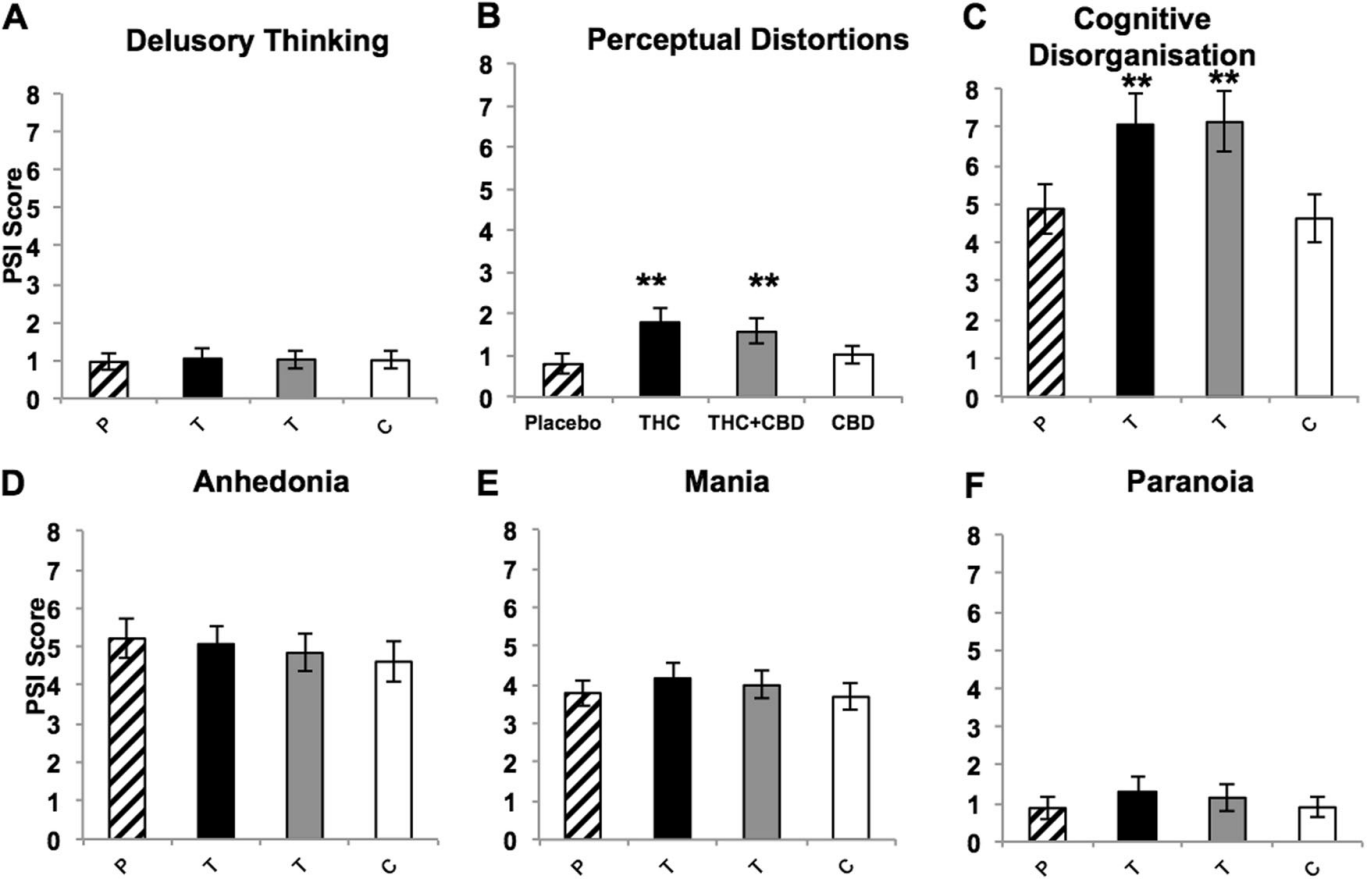
**Effects of THC and CBD alone and in combination, along with placebo, across the whole sample on subscales of psychotomimetic symptoms (PSI)**. THC and THC + CBD increased perceptual distortions (**b**) and cognitive disorganisation (**c**) but not other subscales (**a**; **d**; **e**; **f**). THC = T (Black shading), THC + CBD = T (grey shading), CBD = C, Placebo = P

An interaction between Drug and Subscale was found (*F*_(6,267)_ = 4.881, *p* < 0.001, *ŋ*_*p*_^*2*^ = 0.100) reflecting increased scores following THC and THC + CBD compared to placebo for the subscales of ‘Perceptual Distortion’ (THC: *p* = 0.006; THC + CBD: *p* = 0.005; Fig. 1b) and ‘Cognitive Disorganisation’ (THC: *p* = 0.008; THC + CBD: *p* = 0.004; Fig. 1c). CBD did not elicit change relative to placebo for any of these individual subscales, and no drug effects were found for the remaining subscales of ‘Anhedonia’, ‘Delusory Thinking’, ‘Mania’ and ‘Paranoia’ (Fig. 1).

**Fig. 2:**
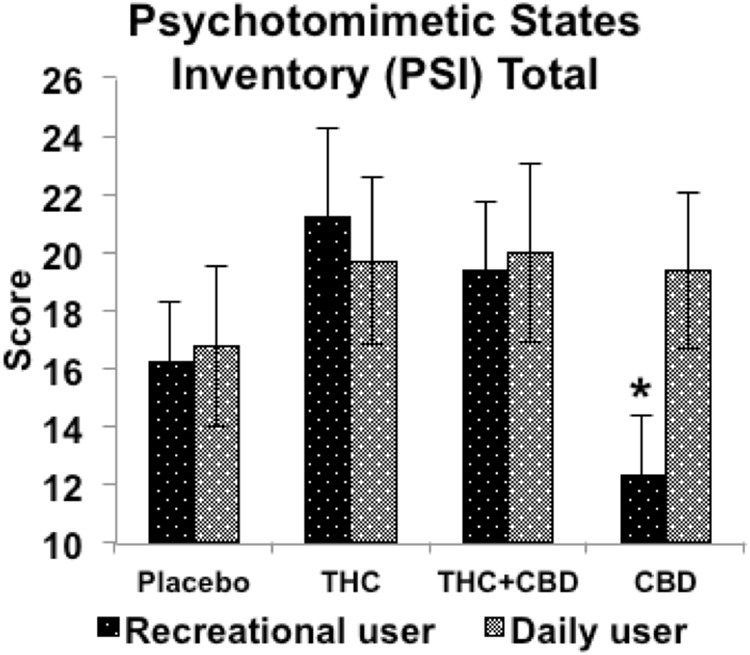
Analysis of effects of THC, CBD, the combination and placebo in low frequency (light) and high frequency (heavy) cannabis users on total psychotomimetic symptom scores

There was also an interaction between Drug and Frequency of use (*F*_(2,105)_ = 3.582, *p* = 0.024, *ŋ*_*p*_^*2*^ = 0.075). Exploration of the interaction showed that light and heavy users had similar responses to THC (*p* = 0.504) and THC + CBD (*p* = 0.977) relative to placebo, but reacted differently to CBD (*p* = 0.005). As shown in Fig. 2, CBD reduced PSI scores relative to placebo in light users (*p* = 0.015), but not in heavy users (*p* = 0.104) as shown in Fig. 2.

Additionally, there was a Schizotypy by Subscale interaction (*F*_(3,150)_ = 6.856, *p* < 0.001, *ŋ*_*p*_^*2*^ = 0.135), as shown in Table 2. Across all drug sessions, high schizotypy volunteers experienced greater PSI scores for ‘Anhedonia’ (*p* < 0.001), ‘Cognitive Disorganisation’ (*p* = 0.001), ‘Mania’ (*p* = 0.004), and ‘Paranoia’ (*p* = 0.007) relative to those with low schizotypy, but no differences were found for ‘Delusory Thinking’ or ‘Perceptual Distortion’.

**Table 2:** Estimated marginal means and standard error (SE) for the Schizotypy by Subscale interaction on the Psychotomimetic States Inventory (PSI), collapsed acrosss drug conditions

	High schizotypy		Low schizotypy	
	*M*	SE	*M*	SE
Delusionary thinking	1.42	0.28	0.59	0.28
Perceptual distortion	1.48	0.30	1.10	0.30
Cognitive disorganisation	7.81	0.73	4.03	0.73
Anhedonia	6.37	0.50	3.48	0.50
Mania	4.77	0.40	3.05	0.40
Paranoia	1.85	0.39	0.27	0.39

Significant main effects were found for the factors of Subscale (*F*_(3,150)_ = 80.254, *p* < 0.001, *ŋ*_*p*_^*2*^ = 0.646) and Schizotypy (*F*_(1,44)_ = 15.271, *p* < 0.001, *ŋ*_*p*_^*2*^ = 0.258).

### Brief psychiatric rating scale (BPRS)

**Fig. 3:**
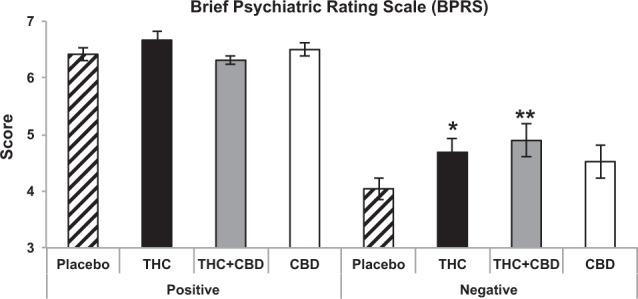
Effects of THC, CBD, the combination and placebo on positive and negative symptoms on the Brief Psychiatric Rating Scale

There was no main effect of Drug. An interaction between Drug and Subscale was found (*F*_(3,132)_ = 3.396, *p* = 0.020, *ŋ*_*p*_^*2*^ = 0.072) as well as a main effect of Subscale, reflecting higher scores for Positive relative to Negative items on the BPRS (*F*_(1,44)_ = 122.149, *p* < 0.001, *ŋ*_*p*_^*2*^ = 0.735). There were no other significant effects or interactions. Exploration of the Drug by Subscale interaction revealed that for Positive items, cannabinoid administration had no effects, but for Negative items both THC (*p* = 0.025) and THC + CBD (*p* = 0.008) increased scores relative to placebo (Fig. 3).

### Prose recall

**Fig. 4:**
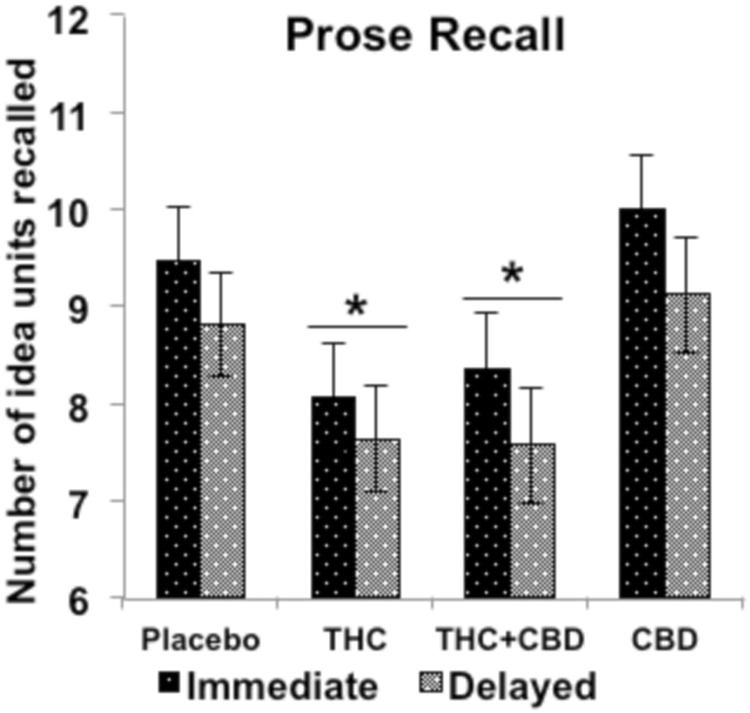
Effects of THC, CBD, the combination and placebo on episodic memory on the prose recall task

There was a main effect of Drug (*F*_(3,132)_ = 4.458, *p* = 0.005, *ŋ*_*p*_^*2*^ = 0.092) which was driven by impairments following THC (*p* = 0.031) and THC + CBD (*p* = 0.024) relative to placebo, whilst CBD had no effect (Fig. 4). A main effect of Delay was also found, reflecting poorer recall at delayed compared to immediate recall (*F*_(1,44)_ = 47.794, *p* < 0.001, *ŋ*_*p*_^*2*^ = 0.521).

### Spatial N-back

**Fig. 5:**
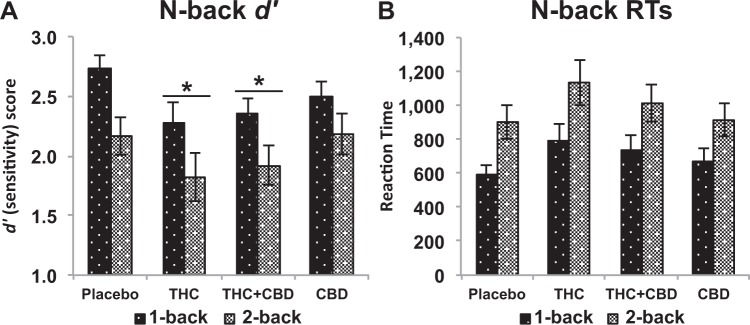
**Working memory.** Effects of THC, CBD, the combination and placebo on working memory in the N back task, for **a** d′ an index of discriminability, and **b** reaction time on the task

There was also a main effect of Drug (*F*_(3,129)_ = 3.421, *p* = 0.019, *ŋ*_*p*_^*2*^ = 0.074), due to a reduction in sensitivity following THC (*p* = 0.012) and THC + CBD (*p* = 0.020) compared to placebo, but no differences for CBD (*p* = 0.532), Fig. 5a. Data for was excluded for one participant on the 1-back task due to an excessively high rate of incorrect responses (69%; chance level: 50%), suggesting they had misunderstood the task instructions. Analysis of *d’* scores revealed a main effect of Load (*F*_(1,43)_ = 16.818, *p* < 0.001, *ŋ*_*p*_^*2*^ = 0.281), attributable to higher *d’* (sensitivity) on the 1-back compared to the 2-back task (Fig. 5a). Analysis of RTs on correct trials revealed a main effect of Load (*F*_(1,43)_ = 18.951, *p* < 0.001, *ŋ*_*p*_^*2*^ = 0.306), due to faster responses on the 1-back compared to the 2-back task (Fig. 5b) but no effect of Drug.

### Fluency

Analysis of exemplars produced on the semantic fluency task revealed a significant effect of Drug (*F*_(3,132)_ = 6.029, *p* = 0.001, *ŋ*_*p*_^*2*^ = 0.121) which was driven by higher scores following THC + CBD (M:19.00, SE: 0.546) compared to placebo (M:16.63, SE: 0.655) (*p* = 0.005) but no differences for THC (M: 17.2 SE:0.68) or CBD (M:14.3 SE:. 0.34) No significant effects emerged for the number of exemplars produced, or errors, on phonological fluency. No effects were found for semantic fluency errors.

### Retain’s trailmaking test

For part A, a significant effect of Drug was found (*F*_(3,132)_ = 4.211, *p* = 0.013, *ŋ*_*p*_^*2*^ = 0.087). This was driven by faster completion following CBD (M: 14.20, SE: 0.47) compared to placebo (M: 15.76, SE: .76) (*p* = 0.045) but no differences for THC or THC + CBD. No significant effects were found for part B, or for part B-part A.

### Correlations

Since the effects of CBD on the PSI were moderated by frequency of cannabis use, correlations were carried out between the effects of CBD on total PSI scores (CBD – placebo) and indices of cannabis use (days of cannabis use per month, years of cannabis used) separately in light (*n* = 24) and heavy users (*n* = 24). No correlations were found in light users. In heavy users, a trend for a positive correlation emerged for years of cannabis use (*r* = 0.434, *p* = 0.034) indicating that a longer history of cannabis use was associated with blunted antipsychotic effects of CBD.

## Discussion

The main findings of this study were an increase in psychotomimetic symptoms following administration of both THC alone and the combination of THC + CBD. Administration of both THC and the combination (THC + CBD) increased negative symptoms on the BPRS, along with perceptual distortions and cognitive disorganisation on the PSI. Lower frequency cannabis users experienced a reduction in psychotomimetic symptoms following CBD alone compared with placebo. Both THC and THC + CBD impaired performance on episodic and working memory tasks. Contrary to hypotheses, CBD did not offset the psychotomimetic effects of THC.

In line with previous studies, we found that THC in the laboratory at a dose of 8 mg inhaled in a Volcano vaporiser increased some psychotic-like symptoms^7,40^. Contrary to predictions, CBD when given concurrently with THC in a 2:1 ratio had no impact on psychotomimetic symptoms, which were still higher than placebo or CBD alone. It has previously been suggested that CBD may ameliorate the harmful effects of THC on psychotic-like symptoms^51^ which has been supported by some empirical evidence^27,30,32^ including two experimental studies (*n* = 6 in a crossover^27^ and *n* = 48 in parallel groups^32^). The latter study^32^ found a reduction in positive psychotic symptoms following intravenous THC, which did not reach significance following pre-treatment of oral CBD. However, a significant protective effects of CBD was found when the authors compared the number of people who met clinically significant psychosis (an increase from baseline of ≥3 points^15^). It may be the case therefore, that CBD is only protective when THC induces a strong psychotic reaction, which might be achieved by using higher doses of THC. In the current study, although THC increased scores on the PSI (perceptual distortion and cognitive disorganisation) and BPRS negative symptoms, it did not increase BPRS positive or PSI paranoia scores at a group level. This in itself is interesting as the same dose and administration route used here has been reliably shown to increase these symptoms in other studies (e.g., ref. ^40^), possible reasons maybe repeating each of these measures in this four-way crossover design. Additionally both our groups were heavier cannabis users than previous studies which have administered similar doses in the lab and tolerance to psychotomimetic effects has been previously shown (D’Souza et al., 2005). Another important consideration is that it is unclear which CBD:THC ratio is most effective for reducing harm^16^. In this study doses of 8 mg THC and 16 mg CBD were chosen to create a CBD:THC ratio of 2:1, reflecting the upper limit (mean + 3 SD) found in high CBD/low THC cannabis preparations^42^. In addition, Englund et al. pre-treated volunteers with oral CBD (600 mg) and before intravenous THC (1.5 mg/kg). These very different doses and routes of administration (which influence absorption and metabolite profiles^52^) may account for our divergent findings. In street cannabis, findings from our own lab did not suggest an acute protective effect of CBD against the psychotomimetic effects of THC^31^ but rather a chronic effect, related to levels in hair^30,51^, which is in accordance with the findings of this current study.

Similarly with cognition, impairments to working and episodic memory were observed following both administration THC and the combination of THC + CBD. Contrary to our predictions, CBD did not offset the effects of THC. This differs from our naturalistic findings where users smoking cannabis with higher levels of CBD resulted in less impairment than those with lower levels^31^. One reason for this difference from naturalistic findings could be other chemicals present in whole plant cannabis material, including terpenes^53^, which do not occur in our synthetically produced cannabinoids. However, interactive effects of CBD and THC on memory function were also reported by Englund et al. in a controlled study of synthetic oral CBD and intravenous THC^32^. Moreover, in the current data we also found that CBD improved facial affect recognition when administered alone, and offset impairments on the same task when co-administered with THC^38^. It is worth noting that the protective effects in CBD that study were of a small effect size, and limited to a single intensity of facial stimuli (40%)^38^. Moreover, CBD was not protective of immediate verbal recall in Englund et al.^32^, and although it showed evidence for a protective effect on delayed recall, these were not supported by a significant condition by group interaction. Thus, taken together, the ability of CBD to protect against THC-induced cognitive impairment may be of a small effect size, and/or influenced by vulnerability factors which have not been considered in research studies to date.

One interesting and unexpected finding was of the greater number of correct exemplars generated following THC + CBD than placebo, but no differences between placebo and THC alone and CBD alone. This is partly in line with previous findings with cannabis users^54^ of increases in divergent thinking following acute cannabis use. This increase occurred only in the group given CBD alongside THC, one possible tentative explanation is that CBD’s pro-cognitive effects combine with the ability of THC to stimulate novel thinking to result in successful task performance.

In this study, no tolerance to the cognitively impairing or psychotomimetic effects of THC was observed for high compared to low frequency users which contrasts with previous studies^8–10^. Differences between these studies could be attributable to dose and/or route of administration (e.g. smoked or intravenous versus vaporised).

Additionally, previous studies suggesting tolerance develops to the acute cognitive impairing effects of THC have generally been small-scale. However, our findings are consistent with a recent study which like ours, used inhalation of THC via a volcano vaporiser^60^. This was a large-scale, cross-over study with 122 participants ranging from daily cannabis users to very infrequent users (as low as once in the last 3 months). Like our present data, they found no evidence of tolerance to the impairing effects of acute THC on neurocognitive function.

Our rationale for including users high and low in schizotypy was that those high in schizotypy may be more vulnerable to the pro-psychotic effects of THC^11–14^. however this hypothesis was not borne out in the data. Additionally we saw no evidence of tolerance to the psychotomimetic effects when THC was administered in this manner in the lab to heavy cannabis users, when compared to lower frequency users, in contrast to previous findings^8^. There may be a variety of reasons for this, including the fact that the current study used a relatively low dose of THC (8 mg) compared to doses estimated in naturalistic studies (~35 mg in the UK^42^; ~32 mg in the Netherlands^60^). The inhalation procedure was standardised, with the aim of controling for dose titration that may occur with cannabis smokers in a naturalistic setting^42,60^. Previous studies investigating associations between schizotypy and acute psychotomimetic effects of cananbis/THC have used naturalistic designs^11,12^ or retrosepctive reports of drug effects^13,14^. It is therefore possible that differences in dose, smoking behaviour and expectancy/recall may have influenced these findings to some extent. A controlled study^15^ reported similar psychotomimetic effects of THC in people diagnosed with schizophrenia and controls.

An interesting finding to emerge from this research was that CBD alone reduced baseline levels of psychotomimetic symptoms in light but not heavy users. Moreover, we found a trend level correlation in heavy users, suggesting that the antipsychotic effects of CBD were increasingly blunted as years of cannabis use increased. This finding is broadly consistent with our previous finding that CBD in hair was associated with fewer psychotic-like symptoms in light but not heavy users^30^. This relates to recent work suggesting that CBD may be a potential treatment in schizophrenia through boosting brain levels of anandamide^33^. Crucially, that study excluded patients with a positive urine screen for cannanabinoids, which might have influenced their antipsychotic response to CBD. The effect of CBD observed in this study was confined only to low frequency users, suggesting a potential tolerance to these effects in higher frequency users, which may have implications for the future use of this compound in the treatment of schizophrenia, which is highly co-morbid with cannabis use disorders^60^. However, further research with repeated dosing of CBD in patients would be needed to support or refute this possibility. Neurobiologically, that these effects were confined to low frequency users fits with research suggesting that high frequency cannabis users have a reduction in anandamide^60^, potentially as a result of chronic cannabis use, which may mean their endocannabinoid systems are less sensitive to exogenous cannabinoids. In addition, a subtle pro-cognitive effect of CBD was observed on the trailmaking task, but only as an increase in psychomotor speed, without any impact on cognitive flexibility, hence this finding should be treated with caution.

This study has several strengths; it used a large sample size in a four-way cross-over in a highly controlled laboratory setting and the use of well-validated tasks. The Volcano

Vaporiser method of administering cannabinoids produces similar plasma and pulmonal THC levels in comparison to smoked cannabis cigarettes^59,60^ and delivers between 80% of the loaded THC^35,36^. Limitations of the current study include not having plasma measures of THC and CBD, therefore we were not able to accurately verify our controlled inhalation procedure, although it has been verified in previous research^40^.

In summary, our study replicated previous findings of a pro-psychotic effect of THC for some psychotic symptoms e.g. negative symptoms, cognitive disorganisation and perceptual distortions and extended these to include a similar effect of a combination when given in a 2:1 ratio (CBD: THC). Cognitive impairments were evident following THC and the combination but no cognitive impairments were observed following CBD alone. CBD was able reduce sub-clinical psychotic-like symptoms in low frequency cannabis users, but not in those using the drug more heavily. This highlights a potentially important area of further research, given the therepeutic potential of CBD for psychosis and the high incidence of cannabis use disorders in this population.
